# Protransition – an online-course for professionals to optimize the health care service for young people with mental illness in transition from adolescence to adulthood

**DOI:** 10.1192/j.eurpsy.2021.1236

**Published:** 2021-08-13

**Authors:** E. König, S. Reetz, U. Hoffmann, J. Fegert

**Affiliations:** Child And Adolescent Psychiatry/psychotherapy, University Hospital Ulm, Ulm, Germany

**Keywords:** transition, E-Learning, adolescence, emerging adulthood

## Abstract

**Introduction:**

Adolescent transitions to adulthood are a vulnerable phase for the development of mental illnesses. Additionally, there are often disruptions in psychiatric care delivery during the transition phase, potentially leading to a considerable treatment delay with a high risk of early chronification. Thus, the health care system and professionals in both child and adolescent psychiatry and adult psychiatry should be given greater consideration to the transition phase.

**Objectives:**

The aim of the project ProTransition is the development of an online course for health care professionals to give in-depth knowledge of “transition psychiatry”, practical guidance and to sensitize them for the special challenges and needs of young adults with mental illness.

**Methods:**

The online-course is being developed at the Department of Child and Adolescent Psychiatry/ Psychotherapy, Ulm and is expected to start in May 2021. It comprises e.g. special psychopathology of emerging adulthood, clinical interventions for adolescents with mental illness or legal aspects. An innovative and multi-didactical approach with specialized texts, case-studies, online-chats and interviews with experts and young people is applied. Additionally, user satisfaction with the online course will be evaluated.

**Results:**

On the basis of the gained experiences, ideas for new transition-psychiatric treatment models will be derived. The accompanying research will point out the status quo and the course-related increasing knowledge of health care professionals regarding transition psychiatry. First results are expected in November 2021.

**Conclusions:**

As transition psychiatry is facing great difficulties and challenges, professionals should be adequately educated. E-Learning offers a flexible and low-level approach to reach a broad target group.

